# Health-related quality of life of Canadian children and youth prenatally exposed to alcohol

**DOI:** 10.1186/1477-7525-4-81

**Published:** 2006-10-13

**Authors:** Brenda C Stade, Bonnie Stevens, Wendy J Ungar, Joseph Beyene, Gideon Koren

**Affiliations:** 1 Department of Paediatrics, St. Michael's Hospital, Toronto, Canada; 2 Sick Kids, Toronto, Canada; 3 Department of Health Policy, Management and Evaluation, University of Toronto, Toronto, Canada

## Abstract

**Background:**

In Canada, the incidence of Fetal Alcohol Spectrum Disorder (FASD) has been estimated to be 1 in 100 live births. Caused by prenatal exposure to alcohol, FASD is the leading cause of neuro-developmental disabilities among Canadian children, and youth. Objective: To measure the health-related quality of life (HRQL) of Canadian children and youth diagnosed with FASD.

**Methods:**

A prospective cross-sectional study design was used. One-hundred and twenty-six (126) children and youth diagnosed with FASD, aged 8 to 21 years, living in urban and rural communities throughout Canada participated in the study. Participants completed the Health Utilities Index Mark 3 (HUI3). HUI3 measures eight health attributes: vision, hearing, speech, ambulation, dexterity, emotion, cognition, and pain. Utilities were used to measure a single cardinal value between 0 and 1.0 (0 = all-worst health state; 1 = perfect health) to reflect the global HRQL for that child. Mean HRQL scores and range of scores of children and youth with FASD were calculated. A one-sample t-test was used to compare mean HRQL scores of children and youth with FASD to those from the Canadian population.

**Results:**

Mean HRQL score of children and youth with FASD was 0.47 (95% CI: 0.42 to 0.52) as compared to a mean score of 0.93 (95% CI: 0.92 to 0.94) in those from the general Canadian population (p < 0.001). Children demonstrated moderate to severe dysfunction on the single-attributes of cognition and emotion.

**Conclusion:**

Children and youth with FASD have significantly lower HRQL than children and youth from the general Canadian population. This finding has significant implications for practice, policy development, and research.

## Background

In Canada, the incidence of Fetal Alcohol Spectrum Disorder (FASD) has been estimated to be 1 in 100 live births [1-3]. Caused by prenatal exposure to alcohol, the disorder is the leading cause of developmental and cognitive disabilities among Canadian children and youth, and its effects are life lasting [1-3].

Fetal Alcohol Spectrum Disorder acknowledges that Fetal Alcohol Syndrome is a continuum, with differing degrees of expression of dysfunction and malformation. The full Fetal Alcohol Syndrome is characterized by a triad of signs: 1) prenatal and or postnatal growth retardation; 2) characteristic facial anomalies including short palpebral fissures, flat philtrum, and thin vermilion border of the upper lip; and 3) central nervous system dysfunction demonstrated by intellectual impairment and/or structural abnormalities, microcephaly, developmental delay, and complex behaviour problems [1-4]. Children with FAS often display characteristics such as extreme hyperactivity, aggressiveness, poor judgment, speech and language difficulties. Other clinical manifestations of FAS may include cardiac anomalies, urogenital defects, skeletal abnormalities, and visual and hearing problems. The term "Fetal Alcohol Effects" (FAE) and more recent diagnostic terms such as "Partial Fetal Alcohol Syndrome (PFAS)", "Alcohol-Related Neurodevelopmental Disorder (ARND)", and "Neurobehavioral Disorder – Alcohol Exposed" are used to describe cases of lesser severity in terms of cognitive function and facial and/or organ anomalies, but often with very serious evidence of neurotoxicity [1-5].

Research on FASD has focused primarily on the physical, cognitive, and behavioral characteristics of the condition [4-10]. The characteristics of FASD described in past research suggest the impact that prenatal exposure to alcohol has on day to day life. However, no study has sought to measure the QOL of victims of FASD, and it remained difficult to obtain a comprehensive description of the overall burden of morbidity resulting from prenatal exposure to alcohol. The current research addressed a gap in knowledge by measuring the quality of life of children and youth with FASD.

### HUI and the concept of quality of life

Quality of Life (QOL) is an inclusive concept incorporating many factors that may affect an individual's life. Experts in the field of QOL have held varying viewpoints on how to define the concept [11-14]. Health-Related Quality of Life (HRQL) has been used to describe the subset of QOL directly related to an individual's health [14]. The HRQL construct can be viewed as a method of translating an individual's experience of his or her illness/disability into a quantifiable outcome.

The conceptual framework for this research is guided by Cadman's [15] concept of HRQL. In this perspective, HRQL focuses on aspects of disease or illness that impact directly on the functioning of an individual in everyday life and impact on his or her ability to live a useful and fulfilling life. Thus, the conceptual focus is on functional capacity rather than performance. The intent is to document the extent to which deficits in health status for each attribute inhibit or prohibit normal functioning rather than report the level at which an individual chooses to function, as would be reflected in a measure of performance [16].

In this study, the Health Utilities Index (HUI) was used to measure HRQL. Although the Health Utilities Index has never been used to measure HRQL in Fetal Alcohol Spectrum Disorder, it has used to examine HRQL of children and youth with other disabilities and illness [17]. Utilizing the HUI with children and youth diagnosed with FASD would allow a more comprehensive description of the overall burden resulting from prenatal exposure to alcohol. Thus, the research objective of this study was to measure the impact that FASD has on the HRQL of children and youth ages 8 to 21 years of age, living in urban and rural communities throughout Canada.

## Methods

### Research design

A cross-sectional research design was used.

### Setting

This study was conducted in urban and rural settings throughout Canada.

### Sample

The study sample included: 1) children and youth diagnosed with FAS or FAE, aged 8 to 21 years, and able to complete an interviewer-administered questionnaire; and their parents (biological, adoptive, or foster) who were currently, living with the child/youth who has FASD, or responsible for the care and welfare of that child.

FASworld Canada, a parent support agency representing of over 11 parent support groups, was contacted and explained the nature of the study. Each group appointed a representative and the group representatives were able to contact 126 potential parent participants, representing 88% of their total support group members who met criteria for the study. Using a standardized explanation, the contact person explained the nature of the study to potential participants and requested permission to give his/her name and address, and telephone number to the researcher. All of the 126 potential parent participants, and 72 of their children were enrolled in the study. The study was approved by the joint university/institutional Research Ethics Board and all participants provided written informed consent.

All children and youth in this study were diagnosed with either FAS or FAE by a pediatrician, a psychiatrist or by a team of professionals at a pediatric/general hospital. The newer diagnostic terms such as ARND, PFFAS and others were not assigned to the children/youth in this study. Thus, the study will specifically refer to the terms "FAS" or "FAE", acknowledging that these 2 terms are diagnostic categories within the continuum of the broader category of "Fetal Alcohol Spectrum Disorder". The children and youth vary in the following characteristics: age, gender, cognitive level, educational levels and abilities, behavioural problems, ethnicity, and age of entry into their current home. Their parents also vary in terms of gender, age, marital status, occupation, education and relationship to the child (adoptive, biological, foster) [18].

### Sample size calculation

A reduction of 0.03 in the HRQL Health Utilities Index score is clinically significant [19-21]. Using data from a previous study [17], where the SD for HRQL utility scores in a similar population of children was 0.21 and assuming an álpha of 0.05 and βeta of 0.20 and a 2 tailed test, it was necessary to interview 99 participants.

### Data collection

#### HRQL study instrument: Health Utilities Index-Mark 3 (HUI 3)

The health utilities index, used to collect data and measure the HRQL, in this study was the Health Utilities Index Mark 3 (HUI3). The HUI3 is the latest of a family of generic systems used to measure health status and assess HRQL. The HUI system is comprised of two complementary components: 1) A multi-attribute health status classification system that is used to describe health status; 2) A multi-attribute utility function that is used to value health status as measured within the corresponding multi-attribute health status classification system [22].

The HUI3 health status classification system measures eight health attributes: vision, hearing, speech, ambulation, dexterity, emotion, cognition, and pain. Each of the 8 health attributes measured in the questionnaire has five or six defined levels, ranging from normal function to severe dysfunction. The comprehensive health status of any child, at any particular time, can be described by a eight-element vector (X1, X2, ... and X8) in which Xi represents the level of the attribute i (1 to 5, or 1 to 6), depending on the number of levels defined for each attribute. The HUI3 describes 972,000 unique health states [22,23].

A utility equation was then applied to the multi-attribute health state description of each participant to reflect the global HRQL for that participant. The HUI3 multi-attribute utility score for a health state is calculated according to the following formula:

u = 1.371 (b1 × b2 × b3 × b4 × b5 × b6 × b7 × b8) – 0.371. Here, "u" represents the utility of a chronic health state on the utility scale where the all-worst health state has a utility of 0.00 and perfect health has a utility of 1.00. The letter "b" represents the single-attribute utility score for a specific attribute at a specific functional level. To arrive at the global health-related quality of life score for each participant, the appropriate utility score "b" for each attribute and functional level are substituted into the formula [22-25].

While the HUI3 has not been used with child and youth with FASD, past research has demonstrated construct, content and face validity, and intra and inter-rater reliability of the HUI3 for use with children and adults living with other disabilities [26-28].

Data elicited from the children and their parents with the interview-administered HUI3 reflected the "usual" health status and HRQL of the children, and were not limited to a specific recall periods such as 1 week, 2 weeks or 4 weeks. The "usual" health format is used in population health surveys or in studies of chronic illness where measuring HQRL in short-term illness, or in response to treatment effects are not the point of interest [23].

Reference group data were obtained from the National Longitudinal Health Survey of Children (NLSC). The NLSC is the first Canada-wide survey of children. Starting in 1994, it has gathered information on children from the general Canadian population. These children and their families have been followed over time, with information on education, health, development, behaviour, friends, activities, and others being accumulated. The HUI3 is used in the health component of the NLSC [29].

### Data analysis

The Shapiro-Wilks test was conducted and demonstrated normality of distribution (Shapiro-Wilks test = 0.9759). HRQL utility scores are continuous measures with interval scale properties. The data were fairly symmetrical and therefore, the best point estimate of the sample is the mean. Thus, the mean and 95% CI were calculated. The statistical significance of the difference in mean overall utility scores (alcohol affected vs. reference children) were assessed using a one sample *t*-test.

In an attempt to determine if severity of illness impacts on quality of life, the mean overall HQRL scores of the FAS group (n = 56) were compared to the FAE group (n = 70) using the *t*-test for independent groups.

A Pearson correlation coefficient and an intra-class correlation coefficient (ICC), calculated by using a two way ANOVA, were used to measure the correlation between the child-parent reports of quality of life.

## Results

### Characteristics of the participants

**Figure 1:**
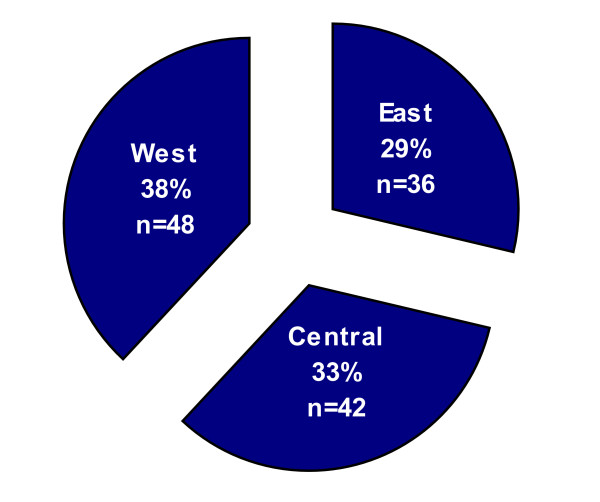
The figure demonstrates an even distribution of participants from Canada's three geographical areas. **West**: Saskatchewan, Alberta, British Columbia, and the Yukon/North West Territories. **Central**: Ontario and Manitoba. **East**: Newfoundland, New Brunswick, Nova Scotia, and Quebec.

One hundred and twenty-six (126) children and their families participated in the study. Seventy-two children (72) and their biological, adoptive, or foster parent (n = 72) completed the HUI3 tool. Fifty-four biological, adoptive or foster parents served as proxy-respondents for 54 children who were unable to complete the HUI3. Reasons for the children not completing the tool were level of disability (n = 42), discomfort of the parent to have the child interviewed (n = 8), or the child was not available for interview (n = 4). All potential parent participants approached agreed to be interviewed. In a two parent home, the parent completing the interview-administered questionnaire was the one who stated they knew the child best. Table 1 presents the gender, diagnosis, ethnicity, age, relationship to the parent, and cognitive level of the 126 children. Figure 1 demonstrates an even distribution of participants from Canada's three geographical areas: West: (Saskatchewan, Alberta, British Columbia, and the Yukon/North West Territories); Central: (Ontario and Manitoba) and East: (Newfoundland and Labrador, New Brunswick, Nova Scotia, and Quebec). Table 2 presents the gender, age, marital status, education, occupation, annual salary, education, and ethnicity of the parents raising the children with FASD.

**Table 1:** Characteristics of the children.

**Characteristics**	**Number**	**(%)**
Gender		
Male	54	42.9
Female	72	57.1
Diagnosis		
FAS	56	44.4
FAE	70	55.6
Ethnicity		
Native	57	45.2
Euro-Canadian	69	54.8
Age		
8–12 years	48	38.1
13–17 years	40	31.7
18–21 years	38	30.2
Age Mean: 14.5 years		
Relationship to Parent		
Biological	14	11.1
Adoptive	70	55.6
Foster	42	33.3
*Cognitive Delay		
Not Delayed	12	9.5
Mild	59	46.8
Moderate	41	32.5
Severe	14	11.2

*Cognitive Delay = Mild: Borderline Range of intellectually deficient level on a standard IQ score (3^rd ^to 8^th ^percentile); Moderate: Intellectually deficient level (0.1 to 2^nd ^percentile); Severe: Intellectually deficient level (less than the 0.1 percentile). Cognitive delay was measured using the Wechsler Intelligence Scale and the Standford-Binet Intelligence Scales.

**Table 2:** HRQL Study – Characteristics of the Parents (n = 126).

**Characteristics**	**Number**	**(%)**
**1. Gender**		
Female	92	73
Male	34	27
**2. Age (in years)**		
< 20	00	00
20 to 30	00	00
20 to 30	22	18
31 to 40	42	33
41 to 50	38	30
51 to 60	24	19
> 60		
**3. Marital Status**		
Married	53	42
Single	28	22
Divorced	26	21
Separated	14	11
Common-in-law	05	04
**4. Occupation**		
Employed Full-Time	54	43
Part-Time	26	21
Unemployed	03	02
Student	16	13
Pension	00	00
Self Employed	09	07
Full-Time Homemaker	18	14
**5. Average annual earned salary**		
less than $10,000	00	00
$10,000 to $20,000	02	02
$20,000 to $30,000	16	13
$30,000 to $40,000	21	17
$40,000 to $50,000	37	29
$50,000 to $60,000	34	27
more than $60,000	16	13
Preferred not to answer	00	00
**6. Education**		
Grade 6 or less	00	00
Grade 7 to completion of Grade 12	37	29
Grade 13 (if applicable and/or Some University or College)	39	31
Completed University or College	48	38
Post Graduate Work	02	02
**7. Ethnicity**		
Euro-Canadian	93	74
Native	33	26

### HRQL scores of children with FASD

The mean HUI3 multi-attribute utility score of children diagnosed with FAS or FAE was 0.47 (95% CI: 0.42 to 0.52). Table 3 presents the mean HRQL scores (n = 126) on each of the 8 health attributes. The children demonstrated moderate to severe dysfunction on the attributes of cognition and emotion, which negatively impacted their overall HRQL scores. The mean HRQL scores of children and youth with FASD was 0.47 (95% CI: 0.42 to 0.52), and compared to a mean score of 0.93 (95% CI: 0.92 to 0.94) in the reference group of children from the general Canadian population. The difference in mean HRQL scores between the two groups was statistically significant (p < 0.001).

**Table 3:** HRQL Study – Single Attribute Utility Score.

**Attribute**	**Scores**	**95% CI**
Vision	1.00	(95% CI: 0.99 to 1.00)
Hearing	0.99	(95% CI: 0.97 to 1.00)
Speech	0.97	(95% CI: 0.96 to 0.98)
Ambulation	1.00	(95% CI: 1.01 to 0.99)
Dexterity	1.00	(95% CI: 0.99 to 1.00)
Emotion	0.76	(95% CI: 0.72 to 0.80)
Cognition	0.83	(95% CI: 0.78 to 0.88)
Pain	1.00	(95% CI: 1.00 to 0.99)

Table 4 presents the frequency distribution of HUI3 attribute levels for all participants. The age range of the sample is very wide both in terms of child development and expected educational and vocational achievements. Although the sample size did not allow statistical analysis, breakdown of scores according to age (Table 5) suggests that age did not impact on the HRQL scores of those with FASD.

**Table 4:** Frequency Distribution (%) of HUI3 Attribute Levels for All Participants (n = 126).

**Level**	**Vision**	**Hearing**	**Speech**	**Ambulation**	**Dexterity**	**Emotion**	**Cognition**	**Pain**
1	126(100)	122(97)	105(83.3)	126 (100)	126 (100)	6(4.8)	16(12.7)	125(99)
2	0(0.0)	3(2.4)	5(4.0)	0 (0.0)	0 (0.0)	16(12.7)	24(19.0)	1 (0.8)
3	0(0.0)	0(0.0)	7(5.6)	0 (0.0)	0 (0.0)	49(38.9)	24(19.0)	0 (0.0)
4	0(0.0)	0(0.0)	6(4.8)	0 (0.0)	0 (0.0)	39(31.0)	34(27.0)	0 (0.0)
5	0(0.0)	0(0.0)	3(2.4)	0 (0.0)	0 (0.0)	16(12.7)	18(14.3)	0 (0.0)
6	0(0.0)	1(0.8)	n/a	0 (0.0)	0 (0.0)	n/a	10(7.9)	n/a
Total	126	126	126	126	126	126	126	126

n/a = not applicable

**Table 5:** Multi-attribute utility scores according to age groups.

**Age**	Mean
8 yr – 10 yr 11 months (n = 29)	0.51 (95% CI: 0.42 to 0.52)
11 yr – 12 yr 11 months (n = 19)	0.49 (95% CI: 0.62 to 0.36)
13 yr – 14 yr 11 months (n = 22)	0.50 (95% CI: 0.61 to 0.39)
16 yr – 17 yr 11 months (n = 18)	0.47 (95% CI: 0.60 to 0.34)
18 yr – 21 years 3 months (n = 38)	0.46 (95% CI: 0.54 to 0.38)

### FAS versus FAE group

Children and youth with Fetal Alcohol Syndrome presented with more organ anomalies, and lower cognitive functioning than children with Fetal Alcohol Effects. Specifically, moderate to severe cognitive delay was found in 71 % of children with FAS as opposed to 32 % in children with FAE. However, parental reports of behavioral problems were similar between the two groups. Given the difference in disabilities, the HRQL scores of children with Fetal Alcohol Syndrome (n = 56) were compared to the HRQL scores of those with Fetal Alcohol Effects (n = 70). Single mean attribute scores of children with FAS and FAE are displayed in Table 6 and demonstrate minimal differences in hearing, speech, emotion, and cognition, and no difference in the remainder of the attributes. While children with FAS demonstrated a lower mean HRQL scores (0.44, 95% CI: 0.37 to 0.52) than those with FAE (0.50, 95% CI: 0.44 to 0.57), the difference was not statistically significant (p > 0.50).

**Table 6:** Single Attribute Utility Score: FAS versus FAE.

**Attribute**	**FAS scores (n = 56)**	**FAE scores (n = 70)**
Vision	1.00 (95% CI: 1.00 to 0.99)	1.00 (95% CI: 1.00 to 0.99)
Hearing	0.99 (95% CI: 1.00 to 0.99)	1.00 (95% CI: 1.00 to 0.99)
Speech	0.97 (95% CI: 1.00 to 0.93)	0.99 (95% CI: 0.99 to 0.98)
Ambulation	1.00 (95% CI: 1.00 to 0.99)	1.00 (95% CI: 1.01 to 0.99)
Dexterity	1.00 (95% CI: 0.99 to 0.98)	1.00 (95% CI: 1.00 to 0.98)
Emotion	0.76 (95% CI: 0.83 to 0.70)	0.75 (95% CI: 0.81 to 0.69)
Cognition	0.81 (95% CI: 0.88 to 0.74)	0.86 (95% CI: 0.92 to 0.80)
Pain	1.00 (95% CI: 1.00 to 0.99)	1.00 (95% CI: 1.01 to 0.99)

### Children – Parents reports of quality of life

The mean total HRQL scores elicited from children who did participate directly in the study (n = 72) were compared to HRQL scores elicited from their parents (n = 72). The children rated their HRQL somewhat higher than their parents with mean scores of 0.56 (SD, 0.24) and 0.47 (SD, 0.27) respectively. A Pearson correlation statistic (r = 0.99, with p <0.001) and an intra-class correlation coefficient (ICC) (0.96 [95% CI: 0.11 to 0.99]) demonstrated that the child and parent HRQL scores were strongly correlated.

## Discussion

This study adds to the body of knowledge on HQRL of children and youth with developmental disabilities. Children/youth who participated in the study consistently demonstrated low HQOL scores, and displayed moderate to severe dysfunction on the attributes of cognition and emotion. HRQL was significantly lower in children and youth with FASD than those from the general Canadian population. In comparison with children who lived with other disabilities or illness, HRQL scores of children with FASD were also extremely low. For example, Saigal et al., [30] measured the HRQL of 141 extremely low birth weight (ELBW) children aged 12 to 16 years, many who lived with significant physical disability such as blindness, cerebral palsy, deafness, and cognitive impairment. In this group of teenagers, the mean HRQL score was 0.87 compared to 0.47 of the sample in the current study. HRQL scores of children with FASD were also much lower than in children who lived with the sequelae of childhood cancers. Specifically, children with FASD reported a lower mean HRQL (0.47) than children who had survived Hodgkin's disease (0.85), brain tumors (0.84), high-risk lymphoblastic leukemia (0.90), Wilm's tumor (0.93) and advanced neuroblastoma (0.87), and childhood cancers in general (0.91) [31-35].

Why children with FAS reported much lower HRQL scores than survivors of childhood cancer or children with significant physical disability needs further investigation. Despite potential sequelae such as learning and behavior problems in children who have been treated for brain tumors, in general, children who have overcome cancer often consider themselves cured, and may in turn perceive their HRQL higher than a child who lives with a chronic disability such as FASD. Why children with physical disabilities score much higher on HRQL is more difficult to understand. Certainly the scores on emotion and cognition in the FAS cohort are much lower than those of other cohorts of children. In the HUI3, the attribute of emotion is more heavily weighted than the other attributes. It should also be considered that children with FASD are often seen as "bad children" [18] whereas children living with a physical disability generally do not carry this stigma. The stigma associated with FASD may in turn impact on HRQL.

Overall, mean HRQL scores of children with FASD are more consistent with other cognitively impaired children and adults, and depressed adults. For example, Glaser et al., [26] found that the mean HRQL score of child survivors of brain tumors was 0.66 with a range of 0.19 to 1.00. A small proportion of these children lived with severe cognitive morbidity, and the utility scores on the attributes of both cognition and emotion were low. Similarly, in patients with Alzheimer's disease (AD), mean HUI3 scores were 0.47 and – 0.23 for questionable AD and terminal AD respectively [28]. Mean HRQL scores of adults with depression were 0.64 to 0.73; 0.55 to 0.63; and 0.30 for mild, moderate, and severe depression respectively [36]. Additional studies measuring HRQL of children with mental illness and cognitive impairment may better reflect the findings of the current study.

### FAS versus FAE

The HRQL scores were similar between children with FAS and FAE. It might be assumed that children with the full syndrome would have a lower quality of life score. Children with the FAE are often more easily integrated into the normal population and appear quite "normal" but often can not perform to societal expectations. For example, in a recent study [37] a boy with FAE indicated how difficult it was when he was unable to meet the expectations of others. Similarly, in an a study by Stade [18], parents of children with FAS and FAE noted that because FAE is often a hidden disability society placed more expectations on these children, than on children who display the physical characteristics of FAS. For example, a father of a teenage boy with FAE stated:

"That's when the hidden disability comes out. Because he looks like he is capable but he isn't. The difficulty is understanding what expectations we can have of him".

The similarity of scores may also be explained by the fact that that there is inconsistency in diagnostic procedures and some individuals diagnosed with FAS in one centre may be diagnosed with FAE in another. In addition, terms used to describe the effects of alcohol exposure on the fetus have changed several times over the last two decades, and remain inconsistent between countries and specific health care institutions.

### Child versus parent reports of HRQL

Wallander et al. [13] noted that there are groups of children who can not provide useful ratings about their HRQL, such as those who are too young, physically ill, or disabled. For some children the only way of obtaining information about their HRQL is by using proxy reporters such as a parent who is asked to reflect on the child's own perception. In this study, 54 children with FASD were not able to participate in the interview primary because of their level of disability. In these circumstances, parents' perspectives were used exclusively as proxy respondents for the child's HRQL.

Although some studies [38,39] have demonstrated poor agreement between parent and child reports, in general, parents have been found to be reliable reporters of their child's quality of life with fair to perfect agreement [40-43]. Agreement among raters may differ as a result of factors such as the child's age, gender, health status and treatment status, and domains measured [41-44].

In keeping with past research, child and parent reports of HRQL were strongly correlated, and demonstrated very good agreement in the current study. The 72 child-parent reports represent children who were capable of participating in the HUI3 and their parents. Thus, we made the assumption that the parent reports of HRQL of the 54 children who did not directly participate, because of their level of disability, were reliable and provided a more accurate picture of HRQL of the total cohort. However, Landgraf [45] caution that high agreement should not suggest that reports from one source is identical to another, and, in this study, valuable perspectives of the children may have been lost when parents served as proxy respondents for their children.

In a study by Stade et al. [37] examining the experience of children and youth with FASD, one major theme that was identified was *establishing a connection with parents*. All of the children with FASD reported that they had a very close relationship with their parents, and some attributed their successes in coping with FASD to their parent(s). These findings suggest the importance of clarifying how the nature of a relationship impacts on the validity of the parent proxy. It is also necessary to clarify if child-parent HRQL scores are similar because the parents know their children's perceptions or because the perception's of the parents have influenced the children over time. More clarification into why children perceive their HQRL somewhat higher than their parents is necessary.

### Study limitations

The study did not draw from a random sample of children and sampling bias is a possibility and must be considered. Not all families of children with FASD join support groups from which the sample was drawn. However, the sampling plan included multiple areas of Canada, and all parents/children agreeing to participate were enrolled. Generalisability of the findings to the larger Canadian population is supported by the heterogeneity of the sample. Individuals residing in institutions such as facilities for disabled children/youth were not included in the sample and it is possible that individuals with severe disability were not well represented in this study. Individuals who were in the judicial system were not included in the study and their inclusion may have lead to somewhat lower HRQL scores. Due to the small sample sizes it was not possible to conduct statistical analysis of HRQL scores according to age groups.

## Implications for practice

HRQL is a most important outcome of treatment for chronic illness and disabilities in childhood. However, in FASD, outcomes related to behavior control have been major treatment goals with little emphasis on quality of life. The negative emotional feelings resulting from the disability may be prevented or reduced if HRQL issues are considered in the treatment of children with FASD. For example, educational programs specific to FASD are needed to help children and youth deal with the demands of the classroom, and to help them feel less different. Such programs which utilize small classroom settings, decreased stimulation and individualized learning strategies have the potential for increasing feelings of success and building self-esteem. Study results demonstrate that children with FASD struggle with depression and anxiety which negatively impacted on the overall HQRL score and on their day-to-day experience of life. This is an opportunity for health professionals to become more involved in developing programs and supports to relieve the children's suffering. All health and social service professionals dedicated to improving the quality of life of disadvantaged children and youth must identify strategies to address the psychosocial needs of those living with Fetal Alcohol Spectrum Disorder.

There is also a need for early diagnosis and early intervention to assist in attenuating the course of the secondary disabilities, including emotional and mental health problems. Early diagnosis can result in greater accessibility to appropriate school programs, greater accessibility to individualized counseling for the child/youth and family, and greater accessibility to specialized programs that can help build the child's self-esteem. Early diagnosis may prevent the "bad child" label that these children often live with. The child, family, and society can come to understand that FASD is a neuro-developmental disorder, and the difficult behaviors displayed by the child are not intentional.

## Implications for research

There is a need for longitudinal studies which determine if quality of life changes over time in children and youth with FASD. There is also a need for research that measures the outcomes of specific treatment programs for these children and youth. The research findings suggest the importance of further clarifying how the nature of the child-parent relationship impact on parent-child agreement of HQOL among children with FASD. When diagnostic terms within the Fetal Alcohol Spectrum become more consistent, it will be important to once more examine if the HRQL of children diagnosed with the full Fetal Alcohol Syndrome differs from those living with other degrees of expression of the disorder. Finally, the HUI3 was found to be a suitable tool for use in this population, and allowed comparisons to children and youth from the general population. However, specific domains such ambulation and vision measured in the HUI3 rarely impact on the quality of life of children/youth with FASD. Results from clinical practice, suggest that other QOL domains such as social interactions and relationships impact on the QOL of children and youth with FASD. Thus, use of multiple tools to elicit QOL, and ultimately, development of a quality of life tool specific to children and youth with FASD is needed.

## Conclusion

This research illustrated that the burden of prenatal exposure to alcohol is profound. The study represents the first of such research on the topic of FASD. The study provides a major contribution to new knowledge and it is anticipated that it will catalyze future research, and changes in practice. Most importantly, it is hoped that the study findings will help children and youth with FASD.

## Competing interests

The author(s) declare that they have no competing interests.

## Authors' contributions

BCS designed the study, collected and analysed the data, and wrote the manuscript.

BS in her role as a PhD committee member, provided guidance throughout the design and conduct of the study. She also reviewed and edited the manuscript. WJU in her role as a PhD committee member, provided guidance throughout the design and conduct of the study. She also reviewed and edited the manuscript. JB in his role as a PhD committee member and an expert statistician, provided guidance in statistical analysis including selection and conduct of statistical tests. GK in his role as Chair of the PhD committee, and an expert on FASD, provided guidance throughout the design and conduct of the study. He also reviewed and edited the manuscript. All authors read and approved the final manuscript.
